# Ursodeoxycholic Acid Use After Bariatric Surgery: Effects on Metabolic and Inflammatory Blood Markers

**DOI:** 10.1007/s11695-023-06581-8

**Published:** 2023-04-25

**Authors:** Maimoena S. S. Guman, Sylke Haal, Yair I. Z. Acherman, Arnold W. L. van de Laar, Max Nieuwdorp, Rogier P. Voermans, Victor E. A. Gerdes

**Affiliations:** 1 Department of Internal and Vascular Medicine, Amsterdam UMC, Amsterdam Gastroenterology Endocrinology Metabolism, University of Amsterdam, Meibergdreef 9, 1105 AZ Amsterdam, the Netherlands; 2 Department of Internal Medicine, Spaarne Gasthuis, Hoofddorp, the Netherlands; 3 Department of Gastroenterology and Hepatology, Amsterdam UMC, Amsterdam Gastroenterology Endocrinology Metabolism, University of Amsterdam, Amsterdam, the Netherlands; 4 Department of Surgery, Spaarne Gasthuis, Hoofddorp, the Netherlands

**Keywords:** Ursodeoxycholic acid, Bariatric surgery, Metabolic markers, Inflammatory markers, Adverse effects, Beneficial effects, Bile acid, Weight loss surgery, Gallstones

## Abstract

**Background:**

In addition to the reduction of symptomatic gallstone disease, ursodeoxycholic acid (UDCA) might also have beneficial metabolic effects after bariatric surgery. We examined the impact of UDCA on liver enzymes, hemoglobin A1c (HbA1c), lipids, and inflammation markers.

**Methods:**

Patients in the UPGRADE trial (placebo-controlled, double-blind) were randomized between UDCA 900 mg daily or placebo pills for 6 months after bariatric surgery. Patients without blood measurements pre- or 6 months postoperatively were excluded. The change in liver enzymes, Hba1c, lipids, and inflammation markers after surgery were compared between the UDCA and placebo group, followed by a postoperative cross-sectional comparison.

**Results:**

In total, 513 patients were included (age [mean ± SD] 45.6 ± 10.7 years; 79% female). Preoperative blood values did not differ between UDCA (*n* = 266) and placebo (*n* = 247) groups. Increase of alkaline phosphatase (ALP) was greater in the UDCA group (mean difference 3.81 U/l [95%CI 0.50 7.12]). Change in other liver enzymes, HbA1c, lipids, and CRP levels did not differ. Postoperative cross-sectional comparison in 316 adherent patients also revealed a higher total cholesterol (mean difference 0.25 mg/dl [95%CI 0.07–0.42]), lower aspartate aminotransferase (mean difference −3.12 U/l [−5.16 – −1.08]), and lower alanine aminotransferase level (mean difference −5.89 U/l [−9.41 – −2.37]) in the UDCA group.

**Conclusion:**

UDCA treatment leads to a higher, but clinically irrelevant increase in ALP level in patients 6 months after bariatric surgery. No other changes in metabolic or inflammatory markers were observed. Except for the reduction of gallstone formation, UDCA has no effects after bariatric surgery.

**Graphical Abstract:**

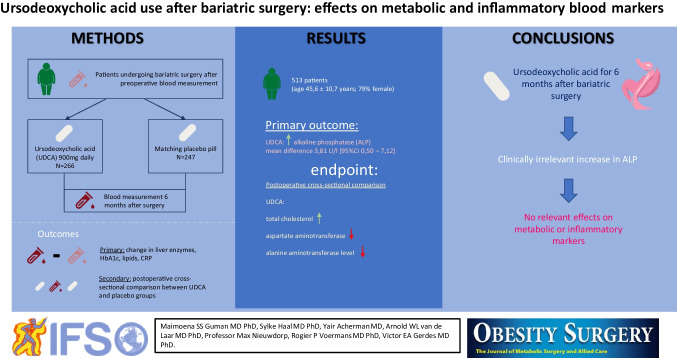

**Supplementary Information:**

The online version contains supplementary material available at 10.1007/s11695-023-06581-8.

## Introduction

Gallstone disease is a common complication after bariatric surgery, the most effective long-term treatment for morbid obesity. Recently, the UPGRADE trial demonstrated that in patients without gallstones before bariatric surgery the prophylactic use of ursodeoxycholic acid (UDCA) for 6 months after surgery reduces the occurrence of symptomatic gallstone disease [1].

UDCA is a naturally occurring bile acid, which is orally prescribed. The most common side effects include diarrhea, nausea, and skin rash. On the other hand, beneficial effects of UDCA treatment such as a reduction in liver enzymes and total cholesterol were reported in patients with primary biliary cirrhosis (PBC) [2, 3]. Accordingly, in addition to the reduction of symptomatic gallstone disease, UDCA treatment might also have beneficial metabolic effects in patients after bariatric surgery. However, little to no evidence is available on the biochemical effects of these drugs in this population.

Furthermore, the exact functions and exerting mechanisms of several bile acids are still not fully understood, but influences on lipids, glucose metabolism, and non-alcoholic fatty liver disease (NAFLD) have been described previously [4]. The gut microbiome in turn has a major influence on bile acid metabolism, especially after bariatric surgery [4]. In fact, a great part of the early effects of bariatric surgery, for example, the almost direct improvement in glucose regulation, has been associated with altered bile acid profiles [5]. In a study by Albaugh et al., increases in UDCA and its glycine and taurine conjugates at 1 month after bariatric surgery led to an increased total bile acid concentration. At 24 months after surgery, increases in bile acid levels were mainly due to increased levels of primary unconjugated bile acids [6]. In addition, studies showed that UDCA has hepatoprotective effects, might change bile acid and lipid metabolism, and improve hepatic insulin sensitivity and immunomodulatory functions [2, 7–13].

The UPGRADE trial, in which patients were randomized to receive either UDCA or placebo, provides us with the possibility to investigate the effects of a high dose of UDCA on liver enzymes, glucose and lipid metabolism, and inflammation. The present study aimed to explore these effects in blood samples of patients before and six months after bariatric surgery.

## Methods

### Study Design and Population

The source population for this cohort study comprised the patients of the UPGRADE trial (Netherlands Trial Register, NL5954), a randomized, multicenter, placebo-controlled, double-blind trial assessing the effect of UDCA on the prevention of symptomatic gallstone disease after bariatric surgery. The protocol, statistical analysis plan, and results of this trial have been published previously [1, 14, 15]. In short, 985 patients aged 18 to 65 years with morbid obesity and an intact gallbladder scheduled to undergo laparoscopic Roux-en-Y gastric bypass (RYGB) or sleeve gastrectomy were included between January 2017 and November 2018. Written informed consent was obtained from all patients. The trial protocol was approved by the institutional review board of the Slotervaart Hospital and Reade (Amsterdam, the Netherlands). Only one of the three centers participating in the UPGRADE trial included a broad regular measurement of blood values 6 months after surgery in their routine follow-up. Hence, for the purpose of the present study, only patients from this center were included. Patients without preoperative or routine postoperative blood measurements 6 months after surgery were excluded thereafter.

### Trial Medication

Patients were randomly assigned to either commercially available UDCA 900 mg daily for 6 months (Ursochol 450 mg tablet; two pills once daily) or matching placebo pills. Patients were instructed to start preferably within 2 weeks, but no later than 8 weeks after surgery. An uninterrupted break of up to 4 weeks was allowed during the treatment course. Patients were allowed to take one pill twice a day or break the pills. Patients, investigators, and treating physicians were all blinded for treatment allocation.

### Data Collection and Procedures

Clinical data including age, gender, weight, body mass index (BMI) before surgery, comorbidities, and medication use were obtained during hospitalization. Blood tests comprising total bilirubin, alkaline phosphatase (ALP), γ-glutamyl transferase (GGT), aspartate aminotransferase (AST), alanine aminotransferase (ALT), total cholesterol, low-density lipoprotein (LDL), high-density lipoprotein (HDL), triglycerides, leukocytes count, C-reactive protein (CRP), hemoglobin A1c (HbA1c), hemoglobin (Hb), platelet count, prothrombin time (PT), total protein, albumin, and calcium were performed as part of regular care at the outpatient clinic before and 6 months after surgery. Parathyroid hormone (PTH) and vitamin D were only measured after surgery. Preoperative blood values were included when obtained up to a maximum of 1 year before bariatric surgery. For postoperative blood values, a window of 4 to 9 months after surgery was applied.

### Outcomes and Definitions

The primary outcomes were liver enzymes (total bilirubin, ALP, GGT, AST, ALT), lipid spectrum (total cholesterol, LDL, HDL, triglycerides), leukocytes, CRP, and HbA1c levels. Secondary outcomes were blood serum levels and counts of Hb, platelets, PT, total protein, albumin, calcium, PTH, and vitamin D. The definitions of diabetes mellitus type 2 (DM2), dyslipidemia, and hypertension were described previously [16, 17]. Adherence in this study was defined as the use of at least 300 pills of trial medication (either UDCA or placebo) within a maximum of 8 months after surgery and a maximum of 4 weeks between the time of the last dose of trial medication taken and the time of blood drawing for the 6-months follow up. The assessment methods of adherence to trial medication in the UPGRADE trial have been described previously [17]. In short, a pill count was performed by the investigators, which was the most decisive for adherence assessment. Furthermore, adherence was self-reported through an online questionnaire completed at 6 weeks and at 6 months after surgery, including verbal reporting at the 6 months follow-up appointment at the outpatient clinic.

### Statistical Methods

Descriptive statistics were used to summarize patient characteristics. First, we calculated the change in blood values (Δ) between 6 months postoperative and preoperative blood values, i.e., the change after bariatric surgery. To investigate the clinical effect of UDCA, we used the unpaired t-test in the main analysis to compare the change in blood values between the group with UDCA treatment and the group with placebo treatment. In order to investigate the actual biochemical effect of UDCA which is not influenced by poor adherence, the second analysis comparing changes in blood values was performed in adherent patients only.

In addition, sensitivity analyses were performed. The first sensitivity analysis was a cross-sectional comparison of blood values at 6 months after bariatric surgery in order to exclude preoperative factors that could influence the outcomes of UDCA treatment. This analysis was repeated in adherent patients only. The second sensitivity analysis was performed in patients without diabetes mellitus to explore the effect of DM2, which was present more often in the placebo group at baseline.

We repeated the analyses in a subgroup with preoperatively elevated liver enzymes and in a subgroup with preoperative dyslipidemia to examine the influences of UDCA use in these specific subgroups. Patients were included in the first subgroup if any of the liver enzymes were elevated (total bilirubin, ALP, GGT, AST, or ALAT). Patients in the second subgroup were included if diagnosed with dyslipidemia but not requiring lipid-lowering drugs before and after bariatric surgery. All additional analyses were also performed using the unpaired t-test.

Finally, regression analysis was used to evaluate whether increased ALP levels were associated with ASAT, ALAT, bilirubin, GGT, calcium, vitamin D, or PTH levels. IBM SPSS statistics (version 26, Armonk, New York) was used and two-sided *P*-values < 0.05 were considered statistically significant.

## Results

**Fig. 1:**
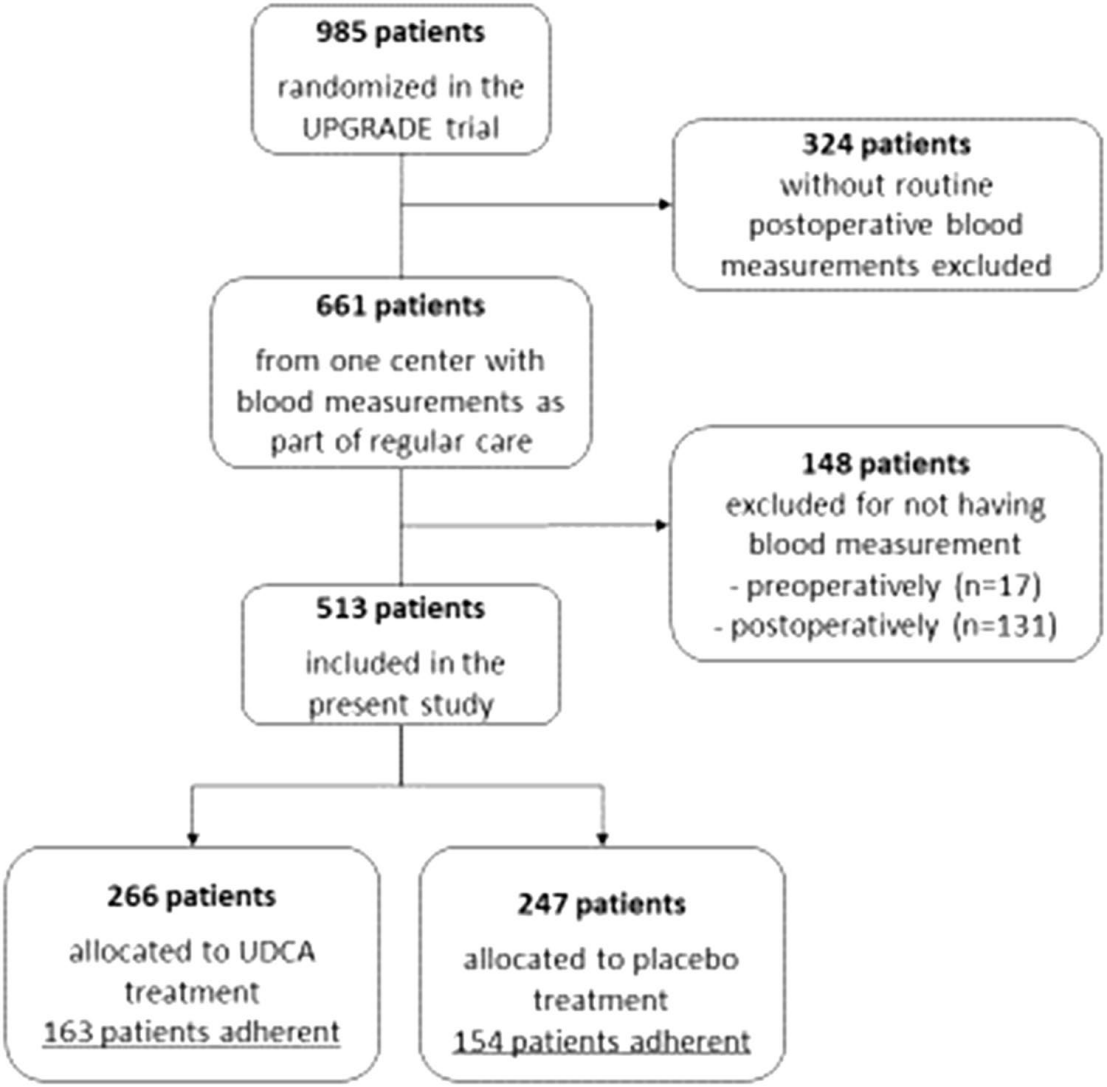
Flowchart of patient selection for the present study

A total of 661 patients were included in the main including center that routinely measured blood values postoperatively. Pre- and postoperative blood measurements were missing in 17 and 131 patients, respectively, leaving 513 patients to be included in the present study (Fig. 1). The mean age (mean ± SD) was 45.6 ± 10.7 years and 79% was female. Compared to the included patients, the group of excluded patients did not significantly differ in age, gender, or BMI before surgery or medication use. As shown in Table 1, 266 patients were allocated to UDCA treatment and 247 to placebo treatment. The preoperative blood values did not differ between the groups. However, DM2 was less prevalent in the UDCA group compared to the placebo group (10.2% versus 17.8%, *p* = 0.01).

**Table 1:** Baseline characteristics and laboratory results for 513 patients treated with trial medication

	Total population *N* = 513	UDCA *N* = 266	Placebo *N* = 247
Patient characteristics			
Age—years	45.6 ± 10.7	46.0 ± 10.7	45.2 ± 10.7
Female gender—n (%)	404 (78.8)	213 (80.1)	191 (77.3)
Weight before surgery—kgs	123.1 ± 17.6	122.7 ± 18.4	123.5 ± 16.7
BMI before surgery—kg/m^2^	39.5 ± 4.2	39.5 ± 4.1	39.5 ± 4.3
Comorbidities—yes (%)			
Hypertension	248 (48.3)	124 (46.6)	124 (50.2)
Dyslipidemia	153 (29.8)	72 (27.1)	81 (32.8)
Diabetes mellitus type 2*	71 (13.8)	27 (10.2)	44 (17.8)
Medication use—yes (%)			
Statin	92 (17.9)	44 (16.5)	48 (19.4)
Oral contraceptive	72 (14.0)	36 (13.5)	36 (14.6)
Laboratory results			
Liver function tests			
Total bilirubin—µmol/l	7.9 ± 3.6	7.9 ± 3.7	8.0 ± 3.4
ALP—U/l	83.7 ± 20.8	84.8 ± 20.8	82.5 ± 20.8
GGT—U/l	33.6 ± 27.4	34.6 ± 30.7	32.4 ± 23.4
AST—U/l	26.4 ± 11.6	25.9 ± 10.7	27.0 ± 12.4
ALT–U/l	33.3 ± 21.6	32.5 ± 19.4	34.3 ± 23.8
Lipid spectrum			
Total cholesterol–mg/dl	4.7 ± 0.9	4.7 ± 1.0	4.6 ± 0.9
LDL–mmol/l	3.0 ± 0.9	3.1 ± 0.9	3.0 ± 0.9
HDL–mmol/l	1.2 ± 0.3	1.2 ± 0.3	1.2 ± 0.3
Triglycerides–mmol/l	1.6 ± 1.3	1.6 ± 1.6	1.5 ± 0.7
Inflammatory parameters			
Leukocytes— × 10^9^/l	7.4 ± 1.8	7.4 ± 1.8	7.3 ± 1.8
CRP—mg/l	7.1 ± 6.7	6.9 ± 6.2	7.4 ± 7.2
Glycemic parameters			
HbA1c—%	5.8 ± 0.8	5.7 ± 0.7	5.8 ± 0.9
Other			
Hemoglobin—mmol/l	8.8 ± 0.8	8.7 ± 0.8	8.8 ± 0.7
Platelets— × 10^9^/l	284.7 ± 61.9	283.4 ± 61.4	286.1 ± 62.5
PT—sec	11.4 ± 2.6	11.4 ± 2.6	11.3 ± 2.6
Total protein—mmol/l	75.0 ± 4.0	75.0 ± 4.1	75.1 ± 3.8
Albumin—g/l	44.4 ± 2.6	44.4 ± 2.7	44.4 ± 2.6

Data are shown as mean ± standard deviation

Abbreviations: *n*, number; *kgs*, kilograms; *BMI*, body mass index; *µmol*, micromole; *l*, liter; *ALP*, alkaline phosphatase; *GGT*, γ-glutamyl transferase; *AST*, aspartate aminotransferase; *ALT*, alanine aminotransferase; *U*, unit; *mg*, milligrams; *dl*, deciliter; *LDL*, low-density lipoprotein; *HDL*, high-density lipoprotein; *mmol*, millimole; *CRP*, C-reactive protein; *HbA1c*, Hemoglobin A1c; *PT*, prothrombin time; *g*, grams

Data was missing in x patients for: CRP, Albumin, ALT: 9 placebo patients and 7 UDCA patients. AST: 12 placebo patients and 10 UDCA patients. Total protein, ALP: 10 placebo patients and 8 UDCA patients. GGT, total cholesterol: 10 placebo patients and 7 UDCA patients. HDL: 9 placebo patients and 12 UDCA patients. LDL: 14 placebo patients and 11 UDCA patients. Triglycerides: 14 placebo patients and 9 UDCA patients. Hemoglobin, platelets, leukocytes: 2 placebo patients and 4 UDCA patients. PT: 11 placebo patients and 8 UDCA patients

^*^The proportion of patients with diabetes mellitus type 2 was greater in patients allocated to placebo treatment, *p* = 0.01

### Changes in Blood Values After Surgery

The changes in blood values after bariatric surgery are shown in Table 2. Compared to the placebo group, the increase of ALP was greater in the UDCA group (mean difference 3.81 U/l [95%CI 0.50–7.12], *p* = 0.02). Changes in the levels of lipids, HbA1c, CRP, leukocytes, and other liver enzymes after bariatric surgery were comparable between groups. The changes for the other blood values (Hb, platelets, PT, total protein, and albumin) did not differ between groups either. Table 3 shows the results of the analysis in 316 adherent patients with blood measurements taken within a maximum of 4 weeks after the last dose of trial medication, with a more pronounced increase of ALP level (mean difference between groups 5.57 U/l [95%CI 1.24–9.89], *p* = 0.01).

**Table 2:** Changes in laboratory results 6 months after bariatric surgery for 513 patients treated with trial medication

	UDCA (*n* = 266)		Placebo (*n* = 247)			
	N =	Mean ± SD	N =	Mean ± SD	Mean difference (95% CI)	*p*-value
Liver function tests						
Total bilirubin—µmol/l	255	1.72 ± 3.57	232	1.58 ± 3.76	0.14 (− 0.51–0.79)	0.67
ALP—U/l	264	9.40 ± 19.85	244	5.59 ± 18.00	3.81 (0.50–7.12)	0.02
GGT—U/l	265	− 16.05 ± 26.95	246	− 12.55 ± 20.47	− 3.50 (− 7.64–0.68)	0.10
AST—U/l	259	− 2.47 ± 12.78	238	− 2.47 ± 14.14	− 0.00 (− 2.38–2.37)	1.00
ALT—U/l	266	− 9.95 ± 29.95	247	− 9.51 ± 28.23	− 0.43 (− 5.49–4.62)	0.87
Lipid spectrum						
Total cholesterol—mg/dl	266	− 0.37 ± 0.74	245	− 0.47 ± 0.70	0.10 (− 0.03–0.22)	0.13
LDL—mmol/l	155	− 0.38 ± 0.61	140	− 0.51 ± 0.54	0.13 (− 0.00–0.26)	0.05
HDL—mmol/l	158	0.12 ± 0.27	144	0.11 ± 0.24	0.01 (− 0.05–0.07)	0.73
Triglycerides—mmol/l	264	− 0.47 ± 1.54	241	− 0.29 ± 0.61	− 0.18 (− 0.39–0.03)	0.08
Inflammatory parameters						
Leukocytes— × 10^9^/l	266	− 0.35 ± 1.63	247	− 0.20 ± 1.52	− 0.15 (− 0.42–0.12)	0.29
CRP—mg/l	265	− 3.71 ± 6.15	244	− 4.07 ± 6.61	0.36 (− 0.75–1.48)	0.52
Glycemic parameters						
HbA1c %	262	− 0.38 ± 0.50	245	− 0.44 ± 0.64	0.05 (− 0.04–0.16)	0.06
Other						
Hemoglobin—mmol/l	266	− 0.34 ± 0.54	247	− 0.33 ± 0.61	− 0.01 (− 0.11 − 0.09)	0.91
Platelets— × 10^9^/l	265	− 16.39 ± 41.17	245	− 20.04 ± 40.34	3.65 (− 3.45–10.75)	0.31
PT—sec	248	0.10 ± 1.21	227	0.10 ± 1.58	0.01 (− 0.25–0.26)	0.96
Total protein—mmol/l	261	− 3.79 ± 3.80	237	− 3.44 ± 3.60	− 0.35 (− 1.00–0.31)	0.30
Albumin—g/l	265	− 1.19 ± 2.84	244	− 0.95 ± 2.85	− 0.24 (− 0.73–0.26)	0.35

Data are shown as mean ± standard deviation

Abbreviations: *µmol*, micromole; *l*, liter; *ALP*, alkaline phosphatase; *GGT*, γ-glutamyl transferase; *AST*, aspartate aminotransferase; *ALT*, alanine aminotransferase; *U*, unit; *mg*, milligrams; *dl*, deciliter; *LDL*, low-density lipoprotein; *HDL*, high-density lipoprotein; *mmol*, millimole; *CRP*, C-reactive protein; *HbA1c*, hemoglobin A1c; *PT*, prothrombin time; *g*: grams

**Table 3:** Changes in laboratory results 6 months after bariatric surgery for 316 patients adherent to trial medication

	UDCA (*n* = 162)		Placebo (*n* = 154)			
	N =	Mean ± SD	N =	Mean ± SD	Mean difference (95% CI)	*p*-value
Liver function tests						
Total bilirubin—µmol/l	157	1.55 ± 3.25	148	1.69 ± 3.70	− 0.14 (− 0.92–0.65)	0.74
ALP—U/l	161	11.31 ± 21.58	152	5.74 ± 16.85	5.57 (1.24–9.89)	0.01
GGT—U/l	162	− 17.52 ± 29.40	154	− 13.42 ± 20.07	− 4.11 (− 9.71–1.49)	0.15
AST—U/l	158	− 3.41 ± 8.93	151	− 3.13 ± 16.37	− 0.28 (− 3.25–2.70)	0.85
ALT—U/l	162	− 12.40 ± 16.84	154	− 10.18 ± 32.75	− 2.22 (− 7.94–3.50)	0.45
Lipid spectrum						
Total cholesterol—mg/dl	162	− 0.40 ± 0.74	153	− 0.48 ± 0.73	0.09 (− 0.08–0.25)	0.29
LDL—mmol/l	96	− 0.39 ± 0.65	91	− 0.56 ± 0.53	0.17 (− 0.00–0.34)	0.05
HDL—mmol/l	99	0.13 ± 0.30	94	0.12 ± 0.26	0.01 (− 0.07–0.09)	0.87
Triglycerides—mmol/l	161	− 0.53 ± 1.89	151	− 0.33 ± 0.66	− 0.21 (− 0.53–0.11)	0.21
Inflammatory parameters						
Leukocytes— × 10^9^/l	162	− 0.34 ± 1.47	154	− 0.17 ± 1.42	− 0.17 (− 0.49–0.15)	0.29
CRP—mg/l	161	− 3.59 ± 6.59	151	− 3.95 ± 6.07	0.36 (− 1.06–1.77)	0.62
Glycemic parameters						
HbA1c %	159	− 0.38 ± 0.51	153	− 0.42 ± 0.62	0.03 (− 0.09–0.16)	0.61
Other						
Hemoglobin—mmol/l	162	− 0.33 ± 0.55	154	− 0.36 ± 0.59	0.03 (− 0.10–0.16)	0.64
Platelets— × 10^9^/l	162	− 12.59 ± 40.79	152	− 17.71 ± 36.44	5.12 (− 3.49–13.73)	0.24
PT—sec	149	0.02 ± 1.50	145	0.03 ± 1.94	− 0.01 (− 0.41–0.39)	0.96
Total protein—mmol/l	159	− 3.91 ± 3.66	149	− 3.27 ± 3.35	− 0.64 (− 1.43–0.15)	0.11
Albumin—g/l	162	− 1.20 ± 2.88	153	− 0.90 ± 2.86	− 0.30 (− 0.94–0.33)	0.35

Data are shown as mean ± standard deviation

Abbreviations: *µmol*, micromole; *l*, liter; *ALP*, alkaline phosphatase; *GGT*, γ-glutamyl transferase; *AST*, aspartate aminotransferase; *ALT*, alanine aminotransferase; *U*, unit; *mg*, milligrams; *dl*, deciliter; *LDL*, low-density lipoprotein; *HDL*, high-density lipoprotein; *mmol*, millimole; *CRP*, C-reactive protein; *HbA1c*, hemoglobin A1c; *PT*, prothrombin time; *g*, grams

### Blood Values After Surgery: Cross-Sectional Comparison

In Table S1, the results of the first sensitivity analysis and the cross-sectional evaluation of postoperative blood values are shown. At 6 months after bariatric surgery, patients treated with UDCA had a significantly higher ALP level compared to patients in the placebo group (mean difference 6.09 U/l (1.72–10.47), *p* < 0.01). The proportion of patients with an elevated ALP level did not significantly differ between groups (24/241 [9.1%] in the UDCA and 18/227 [7.3%] in the placebo group respectively, *p* = 0.48). Furthermore, the total cholesterol level was slightly higher in the UDCA group (mean difference 0.16 mg/dl [95%CI 0.03–0.30], *p* = 0.02). In addition, as can be seen in Table S2, a comparison of the UDCA group and placebo group at six months in 316 adherent patients revealed lower levels of AST (mean difference − 3.12 U/l [− 5.16– − 1.08], *p* < 0.01) and ALT in the UDCA group (mean difference − 5.89 U/l [− 9.41– − 2.37], *p* < 0.01). Only 8 patients had an elevated AST level postoperatively, of whom 3 (1.9%) in the UDCA group and 5 (3.3%) in the placebo group (*p* = 0.44). Similarly, 5 (3.1%) patients in the UDCA group had an elevated ALT level, compared to 11 (7.8%) patients in the placebo group, *p* = 0.06.

### Influence of DM2 and Abnormal Preoperative Blood Values

Subsequently, we performed a second sensitivity analysis in patients without DM2. As can be seen in Table S3, a greater decrease of total cholesterol (mean difference 0.14 mmol/l; 95% CI 0.02–0.27; *p* = 0.03) and LDL (mean difference 0.14 mmol/l; 95% CI 0.01–0.28; *p* = 0.04) was observed in the placebo group, in addition to a greater increase of ALP in the UDCA group (mean difference 4.60 U/l; 95% CI 1.04–8.17; *p* = 0.01).

Last, we analyzed two subgroups. In adherent patients with preoperative elevated liver enzymes, the same responses were observed after surgery in any of the evaluated blood measurements in 64 patients in the UDCA group and 61 patients in the placebo group. However, a cross-sectional comparison at 6 months after surgery revealed higher ALP levels (mean difference 14.48 U/l; 95% CI 3.16–25,80; *p* = 0.01), lower AST levels (mean difference − 4.09; 95%CI − 7.39– − 0.79; *p* = 0.02), and lower ALT levels (mean difference − 5.13; 95%CI − 9.71– − 0.54; *p* = 0.03) in the UDCA group. Next, in 61 patients with untreated dyslipidemia, both the analysis for changes after surgery and cross-sectional analysis showed no differences between the UDCA and placebo group.

### ALP in Relation to Calcium Metabolism

Regression analysis showed that the increase in ALP levels in the total population was correlated with an increase in GGT levels (*r* = 0.22, *p* < 0.01) and a decrease in vitamin D level (*r* =  − 0.10, *p* = 0.03). For patients using UDCA, the increase in ALP was correlated to GGT (*r* = 0.19, *p* < 0.01), but not to vitamin D, PTH, or calcium levels.

At 6 months after surgery, ALP levels in the total population were not correlated to vitamin D, PTH, or calcium levels either. A significant correlation was observed between ALP levels and GT (*r* = 0.24, *p* < 0.01) and bilirubin (*r* = -0.09, *p* = 0.04). However, when analyzed separately, the ALP levels of patients in the UDCA group were significantly correlated to PTH levels (*r* = 0.14, *p* = 0.03) as well as to GGT levels (*r* = 0.18, *p* =  < 0.01). These correlations were even stronger in patients adherent to UDCA treatment (*r* = 0.21, *p* =  < 0.01, and *r* = 0.32, *p* =  < 0.01 for PTH and GGT, respectively). For patients treated with placebo pills, no significant correlations were observed with regard to PTH, vitamin D, or calcium.

## Discussion

In this study, we evaluated the effects of UDCA treatment for six months on liver enzymes, lipid profile, glucose level, and inflammatory markers in patients after bariatric surgery. We found a higher increase in mean ALP level in patients who used UDCA compared to patients receiving placebo treatment. No other significant changes in blood values were observed. Cross-sectional analysis of postoperative blood values did also reveal lower AST and ALT levels, and a slightly higher level of cholesterol in patients who were adherent to UDCA treatment. However, the observed effects of UDCA treatment on liver enzymes, metabolism, and inflammation are minor or absent, and the clinical consequences are negligible. In view of these results, it is confirmed that UDCA treatment in patients after bariatric surgery is safe, but the role in metabolic alterations after surgery seems limited.

To our knowledge, only one study has previously examined the effects of UDCA treatment on clinical blood values in patients after bariatric surgery [18]. In this study, 19 patients used UDCA 3 weeks before surgery, of whom the blood values at 6 months after surgery were compared to the values of 18 patients without treatment prior to surgery. No differences were observed between groups. Other studies focusing on the effects of UDCA treatment other than the prevention or treatment of gallstones were performed in patients with various liver diseases such as chronic hepatitis, PBC, and NAFLD or in mice. In these studies, various effects of UDCA treatment have been described. The studies by Loon et al. and Kim et al. showed a decrease in ALT, AST, and GGT levels after UDCA treatment of obese patients with elevated ALT levels [19, 20]. In PBC patients, some studies described normalization of elevated liver enzymes, whereas other found no effects or elevation of AST [21–25]. In this study, an increase of ALP was observed after bariatric surgery, which was more pronounced in the group treated with UDCA compared to the placebo group. Two other studies also reported higher levels of ALP after bariatric surgery [26, 27]. In these studies, the higher ALP level was thought to be related to bone metabolism, correlated with a high level of PTH. Similarly, ALP levels were related to PTH levels in patients using UDCA in our cohort. Also, postoperative increases in ALP levels were correlated with the decrease in vitamin D levels. However, this correlation was not observed in patients treated with placebo pills. Moreover, this does not explain the higher ALP increase in the UDCA group. In fact, a systematic review by Rudic et al. in PBC patients showed that UDCA significantly decreased ALP levels [25]. Even though ALP was significantly higher in the UDCA group in all the performed analyses, we believe that the clinical consequence of this degree of elevation (9.40 ± 19.85) is clinically irrelevant. With regard to the lipid spectrum, UDCA has been shown to induce a lower cholesterol saturation index and inhibition of lithogenic genes in mice [28]. Moreover, a recent meta-analysis in PBC patients showed that UDCA treatment is associated with a significant lowering of total cholesterol [2].

In contrast to these previous studies, we were not able to detect clinically relevant effects of UDCA use on blood values in this study. Cross-sectional analyses did reveal slightly lower levels of ALT and AST and a marginally higher level of total cholesterol in the UDCA group. Also, the main analysis in which changes in values were compared, showed a greater decrease of AST and ALT and less decrease of total cholesterol in the UDCA group, but these differences did not reach statistical significance. This might be explained by the different populations in our study with substantial changes occurring in the body composition and metabolism after bariatric surgery, especially in the first 6 months. For instance, studies focusing on the underlying mechanisms of action of UDCA showed that a changed bile acid profile and microbiome remodeling might be involved [13, 20]. Since these mechanisms are also affected by the bariatric procedure and subsequent dietary changes, this might interfere with the effects of UDCA. Another explanation for our results might be that out of the different known bile acids, UDCA is less able to influence metabolism (after bariatric surgery) than other bile acids. This was illustrated in the study by Nielsen et al. who showed that UDCA did not affect any plasma hormone concentrations in patients after RYGB, while chenodeoxycholic acid increased plasma concentrations of glucagon-like peptide-1, glucacon, and total bile acids [29].

The strengths of this study include the randomized controlled study design (as part of the UPGRADE trial) and the relatively large sample size. Although power calculations were not performed for the endpoints of the present study, we were not able to detect any additional effects of UDCA. Even if these effects on laboratory markers could be detected in larger study populations, they are probably too small and therefore nog clinically relevant. Furthermore, adherence was assessed in a structured manner, providing the possibility to assess both clinical effects in the entire population and biological effects in patients who actually used UDCA according to prescriptions. However, several limitations should be addressed. First, laboratory measurements were not included in the UPGRADE trial design but were part of regular clinical assessment in the largest participating center, which resulted in missing values and exclusion of results on blood drawn more than 4 weeks after the last dose of trial medication. Second, patients allocated to prophylactic UDCA use were prescribed 900 mg of UDCA daily. This was independent of their body weight before or in the first 6 months after bariatric surgery, whereas UDCA in other conditions such as PBC is prescribed based on body weight. Hence, for some patients, 900 mg might be below the recommended dose at first and might have changed to an appropriate dose or overdose during weight loss. On the other hand, the recommended dose is higher for PBC than for gallstone dissolution. Third, the number of patients known with non-alcoholic fatty liver disease or steatohepatitis in our study population was limited, most probably because these conditions were not structurally recorded in patient’s records. Therefore, we cannot make any statements about the effects of UDCA treatment in this subgroup of patients. However, the analysis in the subgroup of the patient with any of the liver enzymes elevated before surgery did not result in clinically relevant changes either. This is in line with previous studies in patients with NAFLD showing ambivalent effects of UDCA treatment [30]. Last, our study focuses on clinical blood values, which are routinely measured after bariatric surgery as part of the postoperative follow-up care, but the effects of UDCA are probably much more extended. Although our results can be used in clinical practice in case of blood value abnormalities, which may or may not be related to UDCA use, this study lacks data to make statements about the influences of UDCA on bile acid metabolism and other pathways involved in the early beneficial effects observed after bariatric surgery and the prevention of gallstone formation. Extensive studies including histologic assessment of the liver and measurement of serum and fecal bile acids, among other things are needed to assess a possible role of UDCA in these pathways.

In conclusion, UDCA treatment after bariatric surgery does not seem to affect liver function, lipid, glucose, and inflammatory metabolism in a clinically relevant way. Only a limited effect on ALP was noted, which could not be explained by an altered calcium metabolism either. The mechanisms leading to protection against gallstone formation have yet to be identified. Future studies in patients after bariatric surgery should focus on the underlying mechanisms of action in humans and also investigate the effects of treatment with other bile acids, for example, chenodeoxycholic acid, which might be more beneficial to human metabolism.

## Supplementary Information

Below is the link to the electronic supplementary material.

Supplementary file1 (DOCX 54 KB)
