# Supplementary material for: Ursodeoxycholic Acid Use After Bariatric Surgery: Effects on Metabolic and Inflammatory Blood Markers
Source: Obes Surg. 2023 Apr 25;33(6):1773–81. doi: 10.1007/s11695-023-06581-8 (PMC10234851; doi:10.1007/s11695-023-06581-8)
Supplement: Supplementary file 1 — Supplementary file1 (DOCX 54 KB) [file 11695_2023_6581_MOESM1_ESM.docx]

| **Table S1. Laboratory results at 6 months postoperatively for 513 patients treated with trial medication.** | | | | | | |
| --- | --- | --- | --- | --- | --- | --- |
|  | **UDCA (n=266)** | | **Placebo (n=247)** | |  | |
|  | **N =** | **Mean** ± SD | **N =** | **Mean** ± SD | **Mean difference** (95% CI) | **p-value** |
| **Liver function tests** |  | | | | | |
| Total bilirubin - µmol/l | 265 | 9,72 ± 5,07 | 245 | 9,54 ± 4,84 | 0,13 (-0,76 – 1,01) | 0,77 |
| ALP - U/l | 265 | 94,21 ± 25,95 | 245 | 88,11 ± 24,21 | 6,09 (1,72– 10,47) | <0,01 |
| GGT - U/l | 265 | 18,55 ± 17,11 | 247 | 19,86 ± 20,49 | -1,32 (-4,58 – 1,95) | 0,43 |
| AST - U/l | 260 | 23,38 ± 11,81 | 241 | 24,37 ± 9,82 | -0,99 (-2,90 – 0,93) | 0,31 |
| ALT - U/l | 266 | 22,50 ± 24,77 | 247 | 24,79 ± 17,23 | -2,28 (-6,01 – 1,45) | 0,23 |
| **Lipid spectrum** |  | | | | | |
| Total cholesterol - mg/dl | 266 | 4,33 ± 0,77 | 246 | 4,17 ± 0,80 | 0,16 (0,03 – 0,30) | 0,02 |
| LDL - mmol/l | 159 | 2,64 ± 0,71 | 142 | 2,54 ± 0,77 | 0,09 (-0,07 – 0,26) | 0,27 |
| HDL - mmol/l | 161 | 1,34 ± 0,30 | 144 | 1,30 ± 0,27 | 0,04 (-0,02 – 0,11) | 0,18 |
| Triglycerides - mmol/l | 265 | 1,15 ± 0,51 | 243 | 1,20 ± 0,52 | -0,05 (-0,14 – 0,04) | 0,31 |
| **Inflammatory parameters** |  | | | | | |
| Leukocytes - × 10^9^/l | 266 | 7,01 ± 1,82 | 247 | 7,15 ± 1,97 | -0,14 (-0,46 – 0,19) | 0,42 |
| CRP - mg/l | 265 | 3,17 ± 5,58 | 244 | 3,35 ± 4,57 | -0,18 (-1,07 – 0,71) | 0,69 |
| **Glycemic parameters** |  | | | | | |
| HbA1c % | 262 | 5,34 ± 0,45 | 247 | 5,39 ± 0,55 | -0,05 (-0,13 – 0,04) | 0,30 |
| **Other** |  | | | | | |
| Hemoglobin - mmol/l | 266 | 8,39 ± 0,75 | 257 | 8,46 ± 0,77 | -0,07 (-0,20 – 0,07) | 0,32 |
| Platelets - × 10^9^/l | 265 | 267,54 ± 60,06 | 245 | 266,11 ± 62,34 | 1,43 (-9,23 – 12,08) | 0,79 |
| PT - sec | 249 | 11,46 ± 2,16 | 229 | 11,39 ± 1,22 | 0,08(-0,24– 0,39) | 0,64 |
| Total protein - mmol/l | 262 | 71,24 ± 4,01 | 238 | 71,70 ± 4,26 | -0,46 (-1,19 – 0,27) | 0,21 |
| Albumin - g/l | 265 | 43,20 ± 2,55 | 244 | 43,45 ± 2,71 | -0,25 (-0,71 – 0,21) | 0,28 |
| PTH - pmol/l | 252 | 4,88 ± 2,07 | 234 | 4,96 ± 1,92 | -0,08 (-0,43 – 0,28) | 0,67 |
| Calcium - mmol/l | 265 | 2,37 ± 0,10 | 245 | 2,37 ± 0,10 | 0,01 (-0,01 – 0,02) | 0,53 |
| Vitamin D - nmol/l | 265 | 83,25 ± 27,15 | 245 | 80,58 ± 29,53 | 2,67 (-2,26 – 7,60) | 0,29 |
| Data are shown as mean ± standard deviation. Abbreviations: µmol: micromole; l: liter; ALP: alkaline phosphatase; GGT: γ-glutamyl transferase; AST: aspartate aminotransferase; ALT: alanine aminotransferase; U: unit; mg: milligrams; dl: deciliter; LDL: low-density lipoprotein; HDL: high-density lipoprotein; mmol: millimole; CRP: C-reactive protein; HbA1c: Hemoglobin A1c; PT: prothrombin time; g: grams; PTH: parathyroid hormone; pmol: picomole; nmol: nanomole. | | | | | | |

| **Table S2. Laboratory results at 6 months after bariatric surgery for 316 patients adherent to trial medication.** | | | | | | |
| --- | --- | --- | --- | --- | --- | --- |
|  | **UDCA (n=162)** | | **Placebo (n=154)** | |  | |
|  | **N =** | **Mean** ± SD | **N =** | **Mean** ± SD | **Mean difference** (95% CI) | **p-value** |
| **Liver function tests** |  | | | | | |
| Total bilirubin - µmol/l | 159 | 9,68 ± 5,18 | 150 | 9,89 ± 4,99 | -0,21 (-1,35 – 0,93) | 0,71 |
| ALP - U/l | 162 | 96,07 ± 27,61 | 153 | 87,05 ± 24,71 | 9,03 (3,21 – 14,85) | <0,01 |
| GGT - U/l | 162 | 17,47 ± 16,08 | 154 | 19,21 ± 13,91 | -1,75 (-5,08 – 1,59) | 0,30 |
| AST - U/l | 159 | 21,77 ± 7,28 | 153 | 24,90 ± 10,75 | -3,12 (-5,16 – -1,08) | <0,01 |
| ALT - U/l | 162 | 19,96 ± 10,83 | 154 | 25,84 ± 19,87 | -5,89 (-9,41 – -2,37) | <0,01 |
| **Lipid spectrum** |  | | | | | |
| Total cholesterol - mg/dl | 162 | 4,39 ± 0,75 | 153 | 4,14 ± 0,79 | 0,25 (0,07 – 0,42) | <0,01 |
| LDL - mmol/l | 98 | 2,65 ± 0,73 | 92 | 2,46 ± 0,78 | 0,20 (-0,02 – 0,41) | 0,07 |
| HDL - mmol/l | 100 | 1,34 ± 0,30 | 94 | 1,31 ± 0,30 | 0,03 (-0,05 – 0,11) | 0,49 |
| Triglycerides - mmol/l | 162 | 1,18 ± 0,54 | 153 | 1,20 ± 0,54 | -0,02 (-0,14 – 0,10) | 0,80 |
| **Inflammatory parameters** |  | | | | | |
| Leukocytes - × 10^9^/l | 165 | 7,03 ± 1,75 | 160 | 7,01 ± 1,76 | 0,03 (-0,36 – 0,42) | 0,88 |
| CRP - mg/l | 161 | 3,14 ± 5,94 | 151 | 2,89 ± 3,09 | 0,25 (-0,81 – 1,32) | 0,64 |
| **Glycemic parameters** |  | | | | | |
| HbA1c % | 159 | 5,31 ± 0,43 | 154 | 5,43 ± 0,61 | -0,11 (-0,23 – -0,01) | 0,06 |
| **Other** |  | | | | | |
| Hemoglobin - mmol/l | 162 | 8,48 ± 0,72 | 154 | 8,48 ± 0,71 | 0,00 (-0,16 – 0,16) | 0,97 |
| Platelets - × 10^9^/l | 162 | 267,33 ± 60,26 | 152 | 258,29 ± 57,68 | 9,04 (-4,08 – 22,15) | 0,18 |
| PT - sec | 150 | 11,60 ± 2,75 | 146 | 11,50 ± 1,46 | 0,10 (-0,40 – 0,61) | 0,69 |
| Total protein - mmol/l | 160 | 71,07 ± 4,35 | 150 | 71,61 ± 3,99 | -0,54 (-1,48 – 0,39) | 0,26 |
| Albumin - g/l | 162 | 43,11 ± 2,59 | 153 | 43,66 ± 2,64 | -0,55 (-1,13 – 0,04) | 0,07 |
| Data are shown as mean ± standard deviation. Abbreviations: µmol: micromole; l: liter; ALP: alkaline phosphatase; GGT: γ-glutamyl transferase; AST: aspartate aminotransferase; ALT: alanine aminotransferase; U: unit; mg: milligrams; dl: deciliter; LDL: low-density lipoprotein; HDL: high-density lipoprotein; mmol: millimole; CRP: C-reactive protein; HbA1c: Hemoglobin A1c; PT: prothrombin time; g: grams. | | | | | | |

| **Table S3. Subgroup analysis - Changes in laboratory results 6 months postoperatively in 442 patients without DM2 at baseline.** | | | | | | |
| --- | --- | --- | --- | --- | --- | --- |
|  | **UDCA (n=239)** | | **Placebo (n=203)** | |  | |
|  | **N =** | **Mean** ± SD | **N =** | **Mean** ± SD | **Mean difference** (95% CI) | **p-value** |
| **Liver function tests** |  | | | | | |
| Total bilirubin - µmol/l | 230 | 1,69 ± 3,59 | 190 | 1,57 ± 3,43 | 0,12 (-0,55 – 0,80) | 0,72 |
| ALP - U/l | 237 | 10,48 ± 19,82 | 200 | 5,88 ± 17,73 | 4,60 (1,04 – 8,17) | 0,01 |
| GGT - U/l | 238 | -13,73 ± 19,25 | 202 | -10,70 ± 18,96 | -3,03 (-6,63 – 0,56) | 0,10 |
| AST - U/l | 232 | -2,18 ± 13,16 | 197 | -1,32 ± 12,53 | -0,86 (-3,31 – 1,59) | 0,49 |
| ALT - U/l | 239 | -9,15 ± 30,66 | 203 | -8,37 ± 29,39 | -0,78 (-6,42 – 4,86) | 0,79 |
| **Lipid spectrum** |  | | | | | |
| Total cholesterol - mg/dl | 239 | -0,40 ± 0,71 | 201 | -0,54 ± 0,60 | 0,14 (0,02 – 0,27) | 0,03 |
| LDL - mmol/l | 143 | -0,41 ± 0,60 | 123 | -0,55 ± 0,53 | 0,14 (0,01– 0,28) | 0,04 |
| HDL - mmol/l | 146 | 0,12 ± 0,27 | 126 | 0,10 ± 0,24 | 0,02 (-0,05 – 0,08) | 0,62 |
| Triglycerides - mmol/l | 237 | -0,45 ± 1,51 | 198 | -0,26 ± 0,52 | -0,20 (-0,42 – 0,02) | 0,08 |
| **Inflammatory parameters** |  | | | | | |
| Leukocytes - × 10^9^/l | 239 | -0,36 ± 1,66 | 203 | -0,18 ± 1,45 | -0,18 (-0,48 – 0,11) | 0,22 |
| CRP - mg/l | 238 | -3,67 ± 6,24 | 200 | -4,21 ± 6,89 | 0,54 -0,70 – 1,70) | 0,39 |
| **Glycemic parameters** |  | | | | | |
| HbA1c % | 235 | -0,28 ± 0,27 | 201 | -0,27 ± 0,27 | -0,01 (-0,06 – 0,05) | 0,83 |
| **Other** |  | | | | | |
| Hemoglobin - mmol/l | 239 | -0,34 ± 0,54 | 203 | -0,35 ± 0,59 | 0,01 (-0,10 – 0,12) | 0,86 |
| Platelets - × 10^9^/l | 238 | -16,05 ± 41,48 | 201 | -20,38 ± 39,97 | 4,32 (-3,36 – 12,01) | 0,27 |
| PT - sec | 222 | 0,09 ± 0,92 | 187 | 0,13 ± 1,26 | -0,04 (-0,26 – 0,18) | 0,73 |
| Total protein - mmol/l | 234 | -3,83 ± 3,80 | 195 | -3,66 ± 3,45 | -0,17 (-0,86 – 0,53) | 0,63 |
| Albumin - g/l | 238 | -1,24 ± 2,81 | 201 | -1,16 ± 2,72 | -0,07 (-0,60 – 0,45) | 0,79 |
| Data are shown as mean ± standard deviation. Abbreviations: µmol: micromole; l: liter; ALP: alkaline phosphatase; GGT: γ-glutamyl transferase; AST: aspartate aminotransferase; ALT: alanine aminotransferase; U: unit; mg: milligrams; dl: deciliter; LDL: low-density lipoprotein; HDL: high-density lipoprotein; mmol: millimole; CRP: C-reactive protein; HbA1c: Hemoglobin A1c; PT: prothrombin time; g: grams. | | | | | | |
